# Book Reviews

**Published:** 1869-01

**Authors:** 


### BIBLIOGRAPHICAL NOTICES.

*The Missouri Dental Journal* is the title of a new dental monthly, the first number being dated January 1869.

The Editors are Drs. H. Judd, H. S. Chase and W. H. Eames, all members of the Faculty of the Missouri Dental College, in whose hands a sufficient amount of money has been placed by the Missouri State Dental Society to sustain the Journal for one year.

Commencing under such auspicious circumstances, we have no doubt of its success, and if good wishes avail anything we tender ours with the belief that a permanent foundation has been laid for a long career of usefulness. The professional standing of the Editors and Publishing Committee promise that this new Journal will be ably conducted and richly merit a handsome support from the profession.

The first number presents quite a handsome appearance and contains a number of interesting original articles and selections. The subscription price is $3.00 per year.

Some six months ago we received the first number of a new Journal also published in St. Louis, called the *St Louis Dental Journal*, but as this was the first and last which came to us, we are in ignorance as to whether its publication was suspended or not after the first issue.

*Physician's Medical Compend and Pharmaceutical Formulæ.* Compiled by Edward H. Hance. Published by Hance, Griffith & Co., Philadelphia. The contents consist of Preparations aud Formulas, designed especially for the use of Apothecaries and Physicians who dispense medicines, Weights and Measures, Definitions of Terms denoting the Properties of Remedial Agents, A Materia Medica to which is appended the doses, Classification of Remedies Antidotes and Treatment for Poisons, Periods for the Eruptions of the Teeth, Respirations at Various Ages, Average Frequency of the Pulse at Different Ages in Health, &c., making it a very useful pocket companion.

*The Chemical News.*—The American publishers of this valuable journal have inaugurated a new feature in the form of a supplement, added to the English edition, which contains Notices of the current progress of Chemistry and the Physical Sciences, Notices of New Books, Reviews of the Markets, Movements in Trade, etc. This addition is under the editorial charge of Prof. Charles A. Seely. The publishers of the *Chemical News* are W. A. Townsend & Adams, 434 Broome St. New York.

### EDITORIAL DEPARTMENT.

